# The impact of social expectation pressure on turnover intention among Generation Z employees: a psychological mechanism analysis based on social comparison theory

**DOI:** 10.3389/fpsyg.2026.1689646

**Published:** 2026-05-06

**Authors:** Hongda Wang, Jingyi Xiang, Tianwei Xie

**Affiliations:** Graduated School of Business Administration, DongShin University, Naju, Republic of Korea

**Keywords:** digital workplace, Generation Z, social comparison theory, social expectation pressure, turnover intention, upward social comparison

## Abstract

**Introduction:**

In the digital era, Generation Z employees are increasingly influenced by social media driven peer norms. While traditional turnover models focus largely on internal organizational factors, the impact of external digital psychosocial stressors on employee retention remains largely overlooked. Drawing on social comparison theory, this study investigates the relationship between social expectation pressure and turnover intention, with upward social comparison acting as a mediating mechanism.

**Methods:**

A cross-sectional survey was conducted among 542 Generation Z employees working in Chinese e-commerce firms. Validated scales were used to measure social expectation pressure, upward social comparison, and turnover intention. Hierarchical regression, confirmatory factor analysis, Bootstrapping via PROCESS macro, and robustness checks including propensity score matching and multi-group analysis were employed.

**Results:**

Social expectation pressure showed a significant positive association with turnover intention (*β* = 0.324, *p* < 0.001). Upward social comparison partially mediated this relationship (indirect effect = 0.178, 95% CI = [0.111, 0.247]), accounting for 55.6% of the total effect. These statistical relationships remained consistent across different organizational contexts.

**Discussion:**

The study broadens turnover research by introducing social expectation pressure as a relevant external psychosocial factor and confirms the mediating role of upward social comparison in the digital workplace. The findings offer theoretical and practical insights for understanding employee withdrawal behavior in social media saturated environments. Furthermore, they underscore the need for organizations to recognize and manage the social and psychological dynamics that shape the career decisions of Generation Z employees, conceptualizing turnover intention as a key manifestation of these broader career choices.

## 1 Introduction

In the fast-changing digital economy, the e-commerce sector has become a key driver of structural transformation and changes in employment patterns in emerging markets (Zhang, 2021). At the same time, Generation Z employees, those who grew up with the internet, are entering this industry in large numbers. Their unique values, communication preferences, and career expectations are reshaping how organizations operate and manage people (Lestari, 2019).

Although Generation Z shows strong digital adaptability and innovation potential, they also tend to switch jobs frequently in e-commerce companies. This high turnover not only limits the accumulation of human capital but also creates risks for business performance and team stability (Gaan and Shin, 2023). Understanding the psychological factors related to this trend is essential for companies that want to better support and retain this new generation of workers.

While previous studies on employee turnover have largely focused on traditional internal factors such as job satisfaction (Hom et al., 2017; Li et al., 2019), organizational commitment (Joo and Park, 2017; Allen et al., 2010), and leadership style (Hoch et al., 2018), they often overlook the distinct psychological traits of Generation Z. As digital natives, these employees are constantly immersed in social media environments, making them highly susceptible to external digital psychosocial stressors (Wu et al., 2021). The psychological mechanisms linking these digital stressors to organizational behavior are not yet fully understood. To address this research gap, it is crucial to examine the mediating role of upward social comparison. When digital natives observe idealized representations of their peers online, they frequently engage in upward social comparison. This continuous comparative process can foster feelings of relative deprivation and is closely associated with higher turnover intention.

Social expectation pressure refers to the pressure individuals feel from peers in social networks to meet expectations related to career success, lifestyle, and social status (Twenge et al., 2020; Nesi and Prinstein, 2015). Furthermore, social expectation pressure is theoretically distinct from traditional peer pressure or generalized job stress. It originates primarily from curated virtual environments rather than direct workplace interactions and encompasses broader life domains including career success, lifestyle, and social status. This creates a pervasive evaluative burden unique to digital networks (Yang, 2022). For Generation Z, who are highly engaged with social media, this kind of pressure is stronger and more constant (Twenge and Campbell, 2018). Although the current empirical data is drawn from the Chinese e-commerce sector, the intensive use of social media and the resulting social expectation pressure are global phenomena (Ma and Fang, 2024). The psychological dynamics identified here are highly relevant to digital workplaces worldwide where Generation Z forms a growing segment of the labor force.

Social Comparison Theory provides a useful framework for understanding this issue. Proposed by Festinger (1954), the theory suggests that individuals evaluate their views and abilities by comparing themselves to others. This comparison process is strongly associated with their emotions and decision-making behaviors (Buunk and Gibbons, 2007; Suls and Wheeler, 2000). Building on the above analysis, this study aims to explore how social expectation pressure is associated with Generation Z employees’ turnover intention through the process of upward social comparison. To this end, it seeks to answer three key questions:

1) How is social expectation pressure directly associated with the turnover intention of Generation Z employees?
2) What mediating role does upward social comparison play in the relationship between social expectation pressure and turnover intention?
3) Does this mechanism vary across different individual characteristics and organizational contexts?

This study makes several contributions. First, it broadens the perspective on turnover by shifting the focus from purely internal organizational factors to include external digital psychosocial stressors. Recent research suggests that social media environments create new forms of social comparison pressure that influence individuals’ psychological experiences (Arigo et al., 2024; McComb et al., 2023). By applying social comparison theory to the behavior of Generation Z employees, this study extends existing theoretical models to the context of a digital native workforce (Ma and Fang, 2024).

Second, this research highlights social expectation pressure, a relatively underexplored psychological construct emerging in digitally mediated social environments. By examining its role in shaping employees’ perceptions and attitudes, the study provides an improved framework for understanding the behavior of younger employees in contemporary workplaces (Zhang et al., 2024).

Third, by identifying the psychological pathway linking social expectation pressure, upward social comparison, and turnover intention, this study contributes to the literature by clarifying how digitally induced social pressures may translate into organizational outcomes (Huang and Fan, 2022).

## 2 Literature review and theoretical background

In the context of rapid changes in global labor structures and the deepening penetration of digital technologies, Generation Z has become a key part of the workforce in China’s e-commerce companies. At the same time, their high turnover rate has drawn increasing attention from both scholars and practitioners (Gaan and Shin, 2023). Previous studies suggest that traditional models such as job satisfaction and organizational commitment are no longer sufficient to fully explain the turnover behavior of Generation Z employees (Hom et al., 2017; Li et al., 2019). This calls for an update and expansion of existing theoretical frameworks. Beyond structural job factors, are there psychological variables relevant to the digital age that may significantly relate to turnover intention among Generation Z? This study seeks to address that core question.

In this context, social expectation pressure has emerged as a new psychological variable faced by digital natives. Social media frequently promotes peer narratives of success, lifestyle displays, and markers of social status, shaping strong normative expectations among Generation Z (Twenge et al., 2020; Corbí et al., 2025). While some studies have linked this pressure to well-being and anxiety, its role in organizational behavior, particularly in shaping turnover intention, remains under-theorized and lacks empirical testing.

To better understand this mechanism, social comparison theory (Festinger, 1954) offers a useful theoretical lens. According to the theory, when individuals face social evaluation pressure, they tend to assess their own value by comparing themselves with others. This process becomes highly important when objective standards are absent (Suls and Wheeler, 2000). Given the digital and socially sensitive nature of Generation Z, it is reasonable to expect that social expectation pressure triggers upward social comparison, which may in turn increase their intention to leave the organization (Huang et al., 2022).

### 2.1 Turnover intention

Turnover intention refers to an individual’s conscious plan or tendency to leave the organization (Mobley et al., 1979; Tett and Meyer, 1993). In traditional theories, job satisfaction and organizational commitment are seen as key predictors of turnover intention (Price and Mueller, 1986). These models have provided stable prediction tools and have been widely used in various organizational settings.

However, recent studies have shown that as younger generations adopt new work values, face changing social expectations, and consume information in new ways, the explanatory power of traditional models has declined (Seemiller and Grace, 2016; Twenge, 2017). Generation Z often seeks instant feedback, is sensitive to external evaluations, and cares deeply about social performance. As a result, psychological pressures outside the workplace, particularly those from social networks, have become important factors associated with job stability (Zhong et al., 2024).

It is worth noting that while some research has explored the link between social media and job satisfaction or organizational identity (Fazio et al., 2017; Soltis et al., 2013), these studies mostly treat social media as a communication tool. They fail to capture its embedded psychological effects (Chen and Wei, 2020). In fact, social media is more than a platform for sharing information; it creates a space full of social evaluations and peer expectations. Therefore, a more theory-driven approach is needed to understand its relationship with turnover behavior.

### 2.2 Social expectation pressure

Social expectation pressure crystallizes as an implicit manifestation of social norm influence. It defines the psychological burden individuals experience when they perceive a risk of failing to meet the standards upheld by their social group. This pressure originates primarily from peers and is amplified within digital social environments (Piepiora et al., 2024). While this phenomenon shares fundamental logic with traditional peer influence, its scope and intensity have evolved in the digital era through three distinct shifts.

First, the sources of pressure have expanded beyond immediate physical social circles. Digital platforms allow images, text, and behavioral displays to broadcast these expectations to an indefinite audience. Second, the content of these expectations has diversified. It now extends far beyond traditional career achievement to include lifestyle, consumption patterns, physical appearance, and other personal status markers. Third, the nature of exposure has changed. Digital connectivity facilitates a continuous state of evaluation, which creates persistent psychological strain for the individual (McComb et al., 2023).

The social expectation pressure scale developed by Nesi and Prinstein (2015) categorizes this pressure into three dimensions: career success, lifestyle, and social status. This framework offers a robust foundation for structural modeling. However, existing literature primarily links these pressures to mental health outcomes, leaving a gap regarding their impact on organizational behavior (Arigo et al., 2024). This study integrates this concept into turnover research to examine its role as a precursor to psychological distress and to analyze how it interacts with the internal cognitive processes of the employee.

### 2.3 Social comparison theory

Social Comparison Theory suggests that individuals tend to compare themselves with others in order to evaluate their own opinions and abilities, especially when clear standards are lacking (Festinger, 1954). Wills (1981) further distinguished between upward comparison and downward comparison. Upward comparison often leads to negative emotions such as anxiety and dissatisfaction, while downward comparison can help protect self-esteem.

What fundamentally differentiates the social comparison processes of Generation Z from those of older generational cohorts is the nature and scope of the reference group. Previous generations primarily engaged in bounded comparisons with localized peers, such as immediate coworkers or neighbors (Festinger, 1954). In contrast, Generation Z experiences unbounded, continuous, and algorithmic comparisons. Their reference groups are expanded globally through digital platforms, exposing them to a constant stream of highly curated success narratives (McComb et al., 2023). The theoretical rationale for social expectation pressure triggering upward social comparison lies in the psychological need to resolve evaluative uncertainty. When Generation Z employees experience intense social expectation pressure, they feel an acute need to assess their current standing relative to the imposed norms. To do so, they naturally look to the very platforms that generate these expectations. Because social media algorithms often amplify exceptional achievements and idealized lifestyles, these evaluation targets are almost exclusively upward (Chou and Edge, 2012). Therefore, upward social comparison becomes a particularly salient and unavoidable mechanism for this digital native cohort (Primack et al., 2017).

In organizational settings, social comparison has been shown to relate to job satisfaction, perceptions of fairness, and turnover intention (Greenberg et al., 2007; Buunk and Gibbons, 2007). In recent years, the rise of social media has expanded these comparisons beyond closed workplace environments into open digital spaces. Appel et al. (2016) found that upward comparisons on social media platforms are closely linked to increased levels of depression and feelings of relative deprivation. For Generation Z, this mechanism is particularly active, as their strong dependence on digital media has shaped their orientation toward external evaluations (Verduyn et al., 2017).

Based on this, we propose that when Generation Z employees perceive a large gap between themselves and peer standards on social platforms, such as in terms of career success or lifestyle, they are likely to engage in upward comparison. If these comparisons consistently result in negative self-assessment (“I am not as good as others”), they may accumulate feelings of deprivation and self-doubt. Over time, these negative feelings can lead to dissatisfaction with their current work environment and a stronger desire to leave the organization (Cho et al., 2014). Therefore, this study considers upward social comparison as a key mediating mechanism through which social expectation pressure is associated with turnover intention.

### 2.4 Theoretical framework and research hypotheses

Based on Social Comparison Theory, this study develops a theoretical framework linking social expectation pressure, upward social comparison, and turnover intention. The logic of the framework is as follows:

First, Generation Z employees in digital environments face high levels of social expectation pressure from peers. These expectations often focus on career success, lifestyle, and social status. When individuals perceive this pressure, it activates the psychological process of social comparison, particularly upward comparison.

Second, upward social comparison tends to produce negative psychological outcomes. It should be clarified that concepts such as relative deprivation, career-related anxiety, and reduced self-efficacy are presented here as conceptual illustrations of the broader psychological distress caused by upward comparison, rather than as formal variables measured in this structural model. These conceptual outcomes relate to how individuals view their current job and organization, eventually increasing their intention to leave.

**Figure 1:**
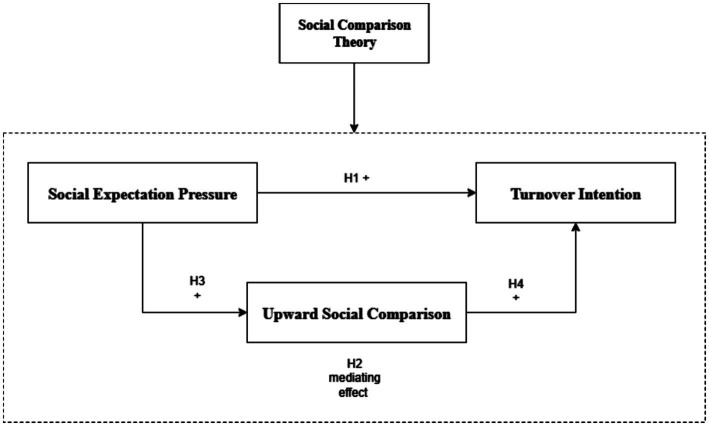
Theoretical framework model diagram.

Third, while upward social comparison serves as a critical pathway, it is unlikely to be the sole mechanism. Social expectation pressure may also directly relate to turnover intention through other unmeasured avenues, such as immediate emotional exhaustion or an impulsive desire to escape high-stress environments. Therefore, a partial mediation model is theoretically more appropriate (Figure 1).

Based on the above theoretical logic, the following hypotheses are proposed:

*H1*: Social expectation pressure is positively related to turnover intention among Generation Z employees.

*H2*: Upward social comparison partially mediates the relationship between social expectation pressure and turnover intention.

*H3*: Social expectation pressure is positively related to upward social comparison.

*H4*: Upward social comparison is positively related to turnover intention.

## 3 Research methodology

### 3.1 Research design and sample selection

This study adopts a cross sectional survey design and focuses on Generation Z employees working in e commerce companies in China. The sample selection criteria required participants to be born between 1995 and 2006, employed in an e commerce company for at least 6 months, using social media for at least 3 h per day, and to have voluntarily agreed to participate in the survey. To ensure sample representativeness, a stratified sampling method was used based on company size including large, medium, and small enterprises, and regional distribution across Tier 1, Tier 2, and Tier 3 cities. The questionnaire was distributed online to employees working in various types of e commerce firms, encompassing B2B and B2C platforms, social commerce, new retail, and livestream selling.

### 3.2 Data collection and quality control

The data were collected through an online questionnaire distributed via the Wenjuanxing platform. To ensure data quality, several control measures were implemented throughout the process. Attention check and reverse coded items were included to identify careless responses. Additionally, a minimum response time of 5 min was set, and responses submitted too quickly were excluded. IP address restrictions were applied to prevent duplicate submissions from the same IP. Logical consistency checks were also utilized by including items with related meanings at different points in the survey. A total of 618 responses were collected. After removing 76 invalid questionnaires, which included 21 with insufficient completion time, 38 with inconsistent logic, and 17 with excessive missing data, 542 valid responses remained. The final valid response rate was 87.7%.

Descriptive statistics for the sample are presented in Table 1. Among the participants, 48.3% were male (*n* = 262) and 51.7% were female (*n* = 280). The age range was 19–30 years, with a mean age of 23.41 years (SD = 2.34). In terms of educational level, 72.5% (*n* = 393) had a bachelor’s degree or above. Regarding work experience, 83.8% (*n* = 454) had 1–3 years of experience, while 16.2% (*n* = 88) had more than 3 years. For job position level, 78.2% (*n* = 424) were front-line employees, and 21.8% (*n* = 118) were middle managers.

**Table 1:** Descriptive statistics of sample characteristics.

Variable	(*N* = 542)	Man (*n* = 262)	Woman (*n* = 280)	Intergroup differences
	*M* (SD) or *n* (%)	*M* (SD) or *n* (%)	*M* (SD) or *n* (%)	*t*/χ^2^
Demographic variables				
Age (years)ᵃ	23.41(2.34)	23.52(2.37)	23.31(2.31)	t = 1.04
Age				X^2^ = 3.45
26–30	89(16.4%)	46(17.6%)	43(15.4%)	
24–25	158(29.2%)	73(27.9%)	85(30.4%)	
22–23	187(34.5%)	89(34.0%)	98(35.0%)	
19–21	108(19.9%)	54(20.6%)	54(19.3%)	
Education level				X^2^ = 3.67
High school or below	23(4.2%)	13(5.0%)	10(3.6%)	
Junior college	126(23.2%)	67(25.6%)	59(21.1%)	
Undergraduate	342(63.1%)	158(60.3%)	184(65.7%)	
Master degree or above	51(9.4%)	24(9.2%)	27(9.6%)	
Marital status				X^2^ = 0.89
Unmarried	487(89.9%)	232(88.5%)	255(91.1%)	
Married	55(10.1%)	30(11.5%)	25(8.9%)	
Work related variables				
Work experience (years)	2.34(1.12)	2.41(1.15)	2.28(1.09)	*t* = 1.35
Less than 1 year	124(22.9%)	56(21.4%)	68(24.3%)	*X*^2^ = 2.78
1–2	193(35.6%)	89(34.0%)	104(37.1%)	
2–3	137(25.3%)	71(27.1%)	66(23.6%)	
More than 3 years	88(16.2%)	46(17.6%)	42(15.0%)	
Position hierarchy				X^2^ = 4.23
Grassroots employees	424(78.2%)	196(74.8%)	228(81.4%)	
Middle managers	118(21.8%)	66(25.2%)	52(18.6%)	
Monthly income (in thousands of yuan)	8.74(3.26)	9.12(3.41)	8.39(3.08)	t = 2.62**
Less than 5,000	89(16.4%)	36(13.7%)	53(18.9%)	X^2^ = 8.45*
5,000–8,000	198(36.5%)	89(34.0%)	109(38.9%)	
8,000–12,000	187(34.5%)	98(37.4%)	89(31.8%)	
Over 12,000	68(12.5%)	39(14.9%)	29(10.4%)	
Job satisfaction	4.12(1.23)	4.18(1.21)	4.07(1.25)	t = 1.04

M = mean; SD = standard deviation; *p < 0.05, **p < 0.01, ***p < 0.001; Job satisfaction (using Smith et al., 1969; 5-point scale, α = 0.86).

### 3.3 Statistical analysis methods

All analyses were conducted using SPSS 28.0 and AMOS 28.0. The analytical procedures began with descriptive statistics to calculate means, standard deviations, and other descriptive indicators for all variables. Reliability and validity analysis followed, where internal consistency was assessed using Cronbach’s *α* and Composite Reliability (CR). Construct validity was tested exclusively through Confirmatory Factor Analysis (CFA) and Average Variance Extracted (AVE), as established scales were utilized. Pearson correlation coefficients were calculated to examine the linear relationships between variables. The Harman’s single factor test and model comparison tests were used to assess the presence of common method bias. For hypothesis testing, hierarchical regression analysis was used to test main effects and mediation effects. While Structural Equation Modeling (SEM) is robust for overall measurement model fit, a regression based approach using the PROCESS macro was selected for hypothesis testing. This approach provides superior computational accuracy for bootstrapping indirect pathways and is highly appropriate for testing mediation models once the measurement model has been validated via CFA. The Bootstrap method was used to assess the significance of mediation effects. Finally, robustness checks such as propensity score matching and subgroup analysis were employed. Propensity score matching was conducted using demographic and job related variables, such as gender, age, and job position level, as covariates to mitigate selection bias. Subgroup analysis was categorized based on company size and city tier to examine contextual boundary conditions.

### 3.4 Measurement of variables and scale selection

To ensure semantic equivalence and cross cultural applicability, all original English scales underwent a rigorous adaptation process. Two bilingual organizational behavior scholars performed the initial translation, and a third independent expert completed the back translation. The Chinese versions were then reviewed by a panel of experts and pilot tested on a small sample prior to formal distribution. Table 2 presents a comprehensive summary of the measurement model’s reliability and convergent validity, including standardized factor loadings, Cronbach’s *α*, Composite Reliability (CR), and Average Variance Extracted (AVE) for all constructs.

**Table 2:** Measurement model reliability and convergent validity.

Construct	Items	Standardized Loadings	Cronbach’s *α*	CR	AVE
Social expectation pressure			0.932		
Career success pressure	SEP1	0.782	0.891	0.885	0.606
	SEP2	0.821			
	SEP3	0.756			
	SEP4	0.798			
	SEP5	0.734			
Lifestyle pressure	SEP6	0.801	0.874	0.887	0.613
	SEP7	0.789			
	SEP8	0.756			
	SEP9	0.823			
	SEP10	0.745			
Social status pressure	SEP11	0.798	0.906	0.886	0.608
	SEP12	0.823			
	SEP13	0.756			
	SEP14	0.789			
	SEP15	0.734			
Upward social comparison	USC1	0.823	0.883	0.932	0.632
	USC2	0.789			
	USC3	0.834			
	USC4	0.756			
	USC5	0.798			
	USC6	0.812			
	USC7	0.745			
	USC8	0.801			
Turnover intention	TI1	0.856	0.911	0.908	0.665
	TI2	0.823			
	TI3	0.798			
	TI4	0.834			
	TI5	0.767			

#### 3.4.1 Social expectation pressure

This study measured social expectation pressure using a revised version of the scale originally developed by Nesi and Prinstein (2015). The scale consists of three dimensions encompassing pressure to achieve career success, pressure to maintain a high quality lifestyle, and pressure related to social status. There are 15 items in total, and sample items include statements like “I feel that my peers expect me to achieve rapid success in my career.” All items were rated using a 7 point Likert scale where 1 means strongly disagree and 7 means strongly agree. Higher scores reflect higher levels of perceived social expectation pressure. The fit indices for the three factor model via CFA were: *χ* 2 /df = 2.14, GFI = 0.92, CFI = 0.94, TLI = 0.93, RMSEA = 0.05, SRMR = 0.04.

#### 3.4.2 Upward social comparison

Upward social comparison was measured using the upward comparison subscale of the Iowa Netherlands Comparison Orientation Measure developed by Gibbons and Buunk (1999). The final version includes eight items, with example items such as “I often compare myself with people who are more successful than I am.” Responses were rated on a 7 point Likert scale where 1 represents strongly disagree and 7 represents strongly agree. Higher scores indicate a stronger tendency toward upward social comparison. Confirmatory factor analysis indicated a good model fit: χ 2/df = 1.96, GFI = 0.94, CFI = 0.96, TLI = 0.95, RMSEA = 0.04.

#### 3.4.3 Turnover intention

Turnover intention was measured using a five item scale developed by Mobley et al. (1978), which has been widely adopted in turnover related studies. Sample statements include “I often think about quitting my current job.” All items were rated on a 7 point Likert scale from 1 for strongly disagree to 7 for strongly agree. Higher scores represent a stronger intention to leave the organization. Confirmatory factor analysis indicated a good model fit: χ 2/df = 1.78, GFI = 0.95, CFI = 0.97, TLI = 0.96, RMSEA = 0.04.

#### 3.4.4 Control variables

Based on previous studies, this research included several control variables that may relate to turnover intention across three distinct categories. Demographic variables included gender, age, educational level, and marital status. Job related variables encompassed years of work experience, job position level, monthly income, and job satisfaction. Lastly, social media use was measured by the average daily time spent on social media and the number of social media platforms used.

## 4 Results

### 4.1 Correlation analysis

The results of the correlation analysis are presented in Table 3. Social expectation pressure was found to be positively correlated with turnover intention (*r* = 0.48, *p* < 0.001) and positively correlated with upward social comparison (*r* = 0.61, *p* < 0.001). In addition, upward social comparison was positively correlated with turnover intention (*r* = 0.52, *p* < 0.001). These findings provide preliminary support for the hypothesized relationships.

**Table 3:** Correlation matrix of key variables.

Variables	1	2	3	4	5	6	7	8
1. Age	1							
2. Gender	−0.027	1						
3. Work experience	0.082	0.718***	1					
4. Job satisfaction	−0.056	0.108*	0.147**	1				
5. Social expectation pressure	0.023	−0.091*	−0.118**	−0.342***	1			
6. Upward social comparison	0.047	−0.073	−0.084	−0.293***	0.612***	1		
7. Turnover intention	−0.019	−0.128**	−0.176***	−0.563***	0.481***	0.521***	1	
8. Duration of social media usage	0.094*	−0.152**	−0.113*	−0.183***	0.314***	0.278***	0.223***	1

*N* = 542. *p < 0.05, **p < 0.01, ***p < 0.001.

### 4.2 Common method bias test

Since all variables were collected through self-reported data from the same source, there is a potential risk of common method bias (CMB). To assess this issue, two commonly used methods were applied:

**Harman’s single-factor test**

An exploratory factor analysis (EFA) was conducted by loading all items from all scales into an unrotated factor solution. As shown in Table 4, the first factor accounted for 36.7% of the total variance, which is below the commonly accepted threshold of 50%. This result suggests that common method bias is not a serious concern in this study.

**Table 4:** Harman’s one-factor test results for common method bias.

Ingredient	Initial eigenvalue	1	2	Extract the sum of squared loads	3	4
	Total	Variance %	Accumulate %	Total	Variance %	Accumulate %
1	12.87	36.77	36.77	12.87	36.77	36.77
2	3.42	9.77	46.54	3.42	9.77	46.54
3	2.16	6.17	52.71	2.16	6.17	52.71
4	1.89	5.4	58.11	1.89	5.4	58.11
5	1.67	4.77	62.88	1.67	4.77	62.88
6	1.43	4.09	66.97	1.43	4.09	66.97
7	1.21	3.46	70.43	1.21	3.46	70.43
8	1.02	2.91	73.34	1.02	2.91	73.34
9	0.89	2.54	75.88			
10	0.78	2.23	78.11			
11	0.69	1.97	80.08			
12	0.62	1.77	81.85			
13	0.57	1.63	83.48			
14	0.52	1.49	84.97			
15	0.48	1.37	86.34			
16	0.44	1.26	87.6			
17	0.41	1.17	88.77			
18	0.38	1.09	89.86			
19	0.35	1	90.86			
20	0.32	0.91	91.77			
21	0.29	0.83	92.6			
22	0.27	0.77	93.37			
23	0.25	0.71	94.08			
24	0.23	0.66	94.74			
25	0.21	0.6	95.34			
26	0.19	0.54	95.88			
27	0.17	0.49	96.37			
28	0.16	0.46	96.83			
29	0.14	0.4	97.23			
30	0.13	0.37	97.6			
31	0.11	0.31	97.91			
32	0.1	0.29	98.2			
33	0.09	0.26	98.46			
34	0.08	0.23	98.69			
35	0.07	0.2	98.89			

Extraction method: Principal Component Analysis; Includes all 35 measurement items (excluding control variables).

**Model fitting comparison**

Table 5 shows that the fit of the forced single factor model is poor (CFI = 0.721), while the fit of the theoretical model (Table 6) is good (CFI = 0.954), and the difference between the two is significant, further confirming that common method bias is not the main problem.

**Table 5:** Fitting index of forced single factor model.

Fitting indicators	Recommendation	Actual value	Judgment
χ^2^	–	2847.63	–
df	–	464	–
χ^2^/df	<3.0	6.14	Unsatisfactory
CFI	>0.90	0.721	Unsatisfactory
TLI	>0.90	0.691	Unsatisfactory
RMSEA	<0.08	0.098	Unsatisfactory
SRMR	<0.08	0.087	Critical
GFI	>0.90	0.742	Unsatisfactory
AGFI	>0.90	0.698	Unsatisfactory

**Table 6:** Mediation analysis results: the role of upward social comparison.

Variable	*β*	se	*t*	*p*
Control variables				
Gender	0.041	0.034	1.21	0.227
Age	−0.047	0.040	−1.18	0.239
Years of work experience	−0.059	0.041	−1.45	0.148
Job satisfaction	−0.194***	0.042	−4.58	< 0.001
Duration of social media usage	0.157***	0.038	4.12	< 0.001
Independent variable				
Social expectation pressure	0.561***	0.037	15.23	< 0.001
Model fit				
*R* ^2^	0.423			
*F*	65.82***			

***p < 0.001.

### 4.3 Measurement model evaluation

To assess the discriminant validity of the three core constructs, a series of confirmatory factor analyses were conducted. The fit indices of the one factor, two factor, and three factor models were compared. Table 7 show the one factor model where all items loaded onto a single factor, the fit indices were *χ* 2 = 2847.63, df = 464, χ 2/df = 6.14, CFI = 0.721, TLI = 0.691, and RMSEA = 0.098. For the two factor model combining social expectation pressure and upward social comparison into one factor, the fit indices were χ2 = 1789.25, df = 463, χ2/df = 3.86, CFI = 0.841, TLI = 0.823, and RMSEA = 0.074. For the three factor model where all constructs were modeled separately, the fit indices were χ2 = 952.14, df = 461, χ2/df = 2.07, CFI = 0.954, TLI = 0.947, and RMSEA = 0.044. As shown in Table 6, the three factor model demonstrated the best fit and performed significantly better than the alternative models where Δχ2 > 10 and *p* < 0.001, indicating good discriminant validity among the three constructs. To provide additional evidence of discriminant validity, the Fornell Larcker criterion was evaluated. Drawing upon the measurement reliability data and the correlation matrix, the square root of the Average Variance Extracted for each construct is strictly greater than its bivariate correlations with any other construct. This finding, combined with the confirmatory factor analysis results, confirms excellent discriminant validity among the key variables.

**Table 7:** Comparison of measurement model fitting degree.

Model	χ^2^	df	χ^2^/df	CFI	TLI	RMSEA	SRMR	AIC	BIC
One-factor model	2847.63***	464	6.14	0.721	0.691	0.098	0.087	2919.63	3124.87
Two-factor model A^1^	1789.25***	463	3.86	0.841	0.823	0.074	0.065	1863.25	2072.34
Two-factor model B^2^	2156.78***	463	4.66	0.798	0.776	0.084	0.076	2230.78	2439.87
Three-factor model	952.14***	461	2.07	0.954	0.947	0.044	0.041	1030.14	1243.56

^1^ Two factor model A combines social expectation pressure and upward social comparison into one factor. ^2^ Two factor model B combines upward social comparison and turnover intention into one factor. ***p < 0.001.

### 4.4 Main effect test

To test Hypothesis 1, a hierarchical regression analysis was conducted using a sample size of 542 to examine the direct relationship between social expectation pressure and turnover intention. Table 8 presents the standardized coefficients for each step of the analysis. Model 1 includes the demographic and job related control variables to predict turnover intention. Model 2 adds the independent variable, social expectation pressure, to assess its direct association with the dependent variable. Model 3 introduces the mediating variable, upward social comparison. Finally, Model 4 includes both the independent and mediating variables simultaneously to evaluate the core relationships. As shown in Table 9, after controlling for demographic and job related variables in Model 2, social expectation pressure was significantly and positively related to turnover intention with a standardized coefficient of *β* = 0.324 and *p* < 0.001. Thus, Hypothesis 1 is supported.

**Table 8:** Hierarchical regression analysis results.

Variables	1	2	3	4
Control variable				
Gender	−0.048 (0.04)	−0.057 (0.04)	−0.068 (0.04)	−0.061 (0.04)
Age	−0.082 (0.06)	−0.061 (0.05)	−0.032 (0.05)	−0.039 (0.05)
Years of work experience	−0.118** (0.05)	−0.091* (0.05)	−0.073 (0.04)	−0.079* (0.04)
Job satisfaction	−0.542*** (0.03)	−0.461*** (0.03)	−0.408*** (0.03)	−0.362*** (0.03)
Duration of social media usage	0.113** (0.04)	0.082* (0.04)	0.064 (0.04)	0.071* (0.04)
Independent variable				
Social expectation pressure		0.324*** (0.04)	0.179*** (0.04)	0.124** (0.04)
Mediating variable				
Upward social comparison			0.312*** (0.04)	0.283*** (0.04)
*R* ^2^	0.356	0.412	0.467	0.489
Δ*R*^2^		0.056***	0.055***	0.022***
*F*	58.73***	62.41***	59.87***	54.23***

*N* = 542. All reported *β* values are standardized coefficients. ***p < 0.001, **p < 0.01, *p < 0.05.

**Table 9:** Bootstrap mediation analysis results.

Effect type	Effect size	Boot SE	Boot LLCI	Boot ULCI
Direct effect	0.142**	0.048	0.047	0.237
Indirect effect	0.178***	0.034	0.111	0.247
Total effect	0.320***	0.041	0.239	0.401
Specific path decomposition				
SEP → USC	0.561***	0.037	0.488	0.634
USC → TI	0.318***	0.052	0.215	0.421
SEP → USC → TI	0.178***	0.034	0.111	0.247

Set the resampling frequency to 5,000 times, SEP = social expected stress; USC stands for Upward Social Comparison; TI = intention to resign, Boot LLCI and Boot ULCI are the lower and upper limits of the 95% confidence interval, respectively. **p < 0.01, ***p < 0.001.

### 4.5 Mediation effect test

To examine the mediating role of upward social comparison, both stepwise regression analysis and the Bootstrap method were employed. First, the relationship between social expectation pressure and the mediating variable representing upward social comparison was tested. As shown in Table 6, social expectation pressure demonstrated a positive association with upward social comparison where β = 0.561 and p < 0.001, supporting Hypothesis 3. Next, the link between upward social comparison and turnover intention was examined. The results in Table 9 indicate that upward social comparison was positively related to turnover intention where β = 0.318 and p < 0.001, providing support for Hypothesis 4.

To further verify the mediating relationship, the Bootstrap method was used via the PROCESS macro. Table 9 results showed that the indirect relationship between social expectation pressure and turnover intention through upward social comparison was statistically valid with an indirect estimate of 0.178 and a 95% confidence interval ranging from 0.111 to 0.247, which securely excludes zero. This indirect path accounted for 55.6% of the total relationship calculated as 0.178 divided by 0.320, indicating that upward social comparison partially mediates the association between social expectation pressure and turnover intention. Since the direct relationship remained statistically valid with a direct estimate of 0.142 and a 95% confidence interval ranging from 0.047 to 0.237 excluding zero, the mediation was partial rather than full. Thus, Hypothesis 2 is partially supported.

### 4.6 Robustness checks

To ensure the robustness of the findings, two additional analyses were conducted:

To account for potential confounding effects of individual characteristics, propensity score matching was performed to compare employees with high versus low levels of social expectation pressure. A median split on the overall social expectation pressure score was utilized as the cut off criterion to define the treatment and control groups. Participants scoring above the median were assigned to the high pressure group, while those scoring below or equal to the median were assigned to the low pressure group. The propensity score estimation included relevant covariates such as gender, age, educational level, work experience, job position level, and monthly income. As shown in Table 10, after controlling for these personal differences, employees in the high pressure group reported significantly higher levels of turnover intention with a mean of 4.82 than those in the low pressure group with means ranging from 3.14 to 3.21. The difference ranged from 1.61 to 1.68 units and was statistically significant where *p* < 0.001, suggesting a robust positive association between social expectation pressure and turnover intention.

**Table 10:** Propensity score matching results.

Matching method	Processing group mean	Control group mean	ATT	SE	*t*	*p*
Nearest-neighbor matching (1:1)	4.82	3.14	1.681***	0.21	8.00	<0.001
Radius matching (*r* = 0.05)	4.82	3.21	1.609***	0.19	8.47	<0.001
Kernel matching	4.82	3.16	1.662***	0.20	8.30	<0.001
Original regression coefficient	–	–	1.721***	0.18	9.56	<0.001

***p < 0.001.

To further test contextual robustness, the total sample was divided into two groups based on company size including large enterprises with a sample of 298 and small and medium enterprises with a sample of 244. Firm size was operationalized through a self reported categorical question in the survey. Participants were asked to classify their employing organization by selecting from predefined categories such as large enterprise, medium enterprise, or small enterprise. For the purpose of this subgroup analysis, responses were aggregated into two distinct categories representing large enterprises and small and medium enterprises. Separate path analyses were conducted for each group. As shown in Table 11, the direction and significance of the path coefficients were consistent across both subsamples. *Z* tests comparing the path coefficients between groups showed no significant differences where *p* > 0.05, suggesting that the findings are robust across organizational contexts.

**Table 11:** Robustness test results by firm size subsamples.

Path relationship	Large enterprise (n = 298)	Small and medium-sized enterprises (n = 244)	Z	p
Social expectation pressure → Turnover intention	0.298***	0.347***	−1.43	0.153
Social expectancy pressure → upward social comparison	0.578***	0.541***	1.28	0.201
Upward social comparison → Turnover intention	0.321***	0.306***	0.52	0.603
Indirect effect	0.186***	0.166***	0.73	0.465

***p < 0.001.

## 5 Discussion

### 5.1 Key research findings

This study provides a comprehensive examination of the psychological drivers behind the career decisions of Generation Z employees. By integrating social expectation pressure and upward social comparison into a single framework, we offer a new understanding of how the digital social environment interacts with professional stability. Our analysis moves beyond traditional turnover models that focus on internal organizational factors to reveal how external social benchmarks frame employee intentions.

First, our results confirm a positive association between social expectation pressure and turnover intention where *β* = 0.324 and *p* < 0.001. This finding provides a necessary update to classical turnover theories, such as the model proposed by Mobley et al. (1978), which primarily emphasized job satisfaction and internal utility. While traditional models suggest that employees leave because of dissatisfaction with tangible job attributes, our data shows that even when job satisfaction is controlled for, external social pressure remains a vital driver of turnover. This suggests that for Generation Z, the boundary between their private social life and professional identity is increasingly blurred (Twenge and Campbell, 2018). The pressure to meet perceived social standards of success acts as a persistent psychological weight that prompts employees to seek alternative career paths that might better align with these external expectations (Huang and Fan, 2022; Sender and Korzynski, 2019).

Second, the identification of upward social comparison as a mediator provides a deeper insight into the internal cognitive process of employees (Smith et al., 2012). The results supporting Hypothesis 3 and Hypothesis 4 show that social pressure does not lead to turnover in a vacuum; rather, it functions by triggering a comparison process. When employees are exposed to the curated successes of their peers on digital platforms, they engage in upward comparisons that often result in feelings of relative deprivation (Karacay et al., 2022). This cognitive gap explains why the mediation effect is so prominent, accounting for 55.6 percent of the total relationship. Unlike prior studies that treated social media usage as a mere distraction, our findings demonstrate that the content of these digital interactions creates a comparative benchmark that directly devalues an employee’s current professional standing (Faelens et al., 2021).

Finally, our robustness checks through propensity score matching and firm size subsample analysis offer important evidence regarding the universality of this mechanism. By using propensity score matching to control for individual differences such as income and experience, we confirmed that the high pressure group consistently reported higher turnover intentions where *p* < 0.001. Furthermore, the multi group analysis showed that the model holds true across both large enterprises and small and medium sized firms with no significant differences in path coefficients where *p* > 0.05. This demonstrates that the psychological impact of social expectation pressure is a pervasive phenomenon among Generation Z workers, regardless of the specific organizational setting or company resource levels (Arigo et al., 2024). This finding advances the literature by showing that the digital social landscape creates a standardized set of pressures that transcend traditional workplace boundaries.

### 5.2 Theoretical contributions

This work contributes to the literature by supplementing the conceptual scope of turnover research. While prior literature focused on internal job attributes or external economic conditions, our results suggest that psychosocial factors originating from the digital social landscape play a vital role (Huang and Fan, 2022; Zhang et al., 2024). By integrating social expectation pressure into the turnover literature, we provide a supplementary lens for interpreting how digital social norms influence career decisions. This does not replace existing models but rather offers a more comprehensive view of the factors driving employee retention in the modern era.

Furthermore, this study demonstrates the utility of social comparison theory in organizational behavior. Although this theory is well established in studies of wellbeing or anxiety, it is rarely applied to the context of turnover (McComb et al., 2023). Our results show that upward comparison is more than a momentary emotional reaction; it carries implications for long term career stability. This demonstrates that social comparison theory offers a relevant framework for understanding the digital workplace where visibility is high.

Our analytical framework also offers a fresh perspective for future research. Traditional studies usually limited comparison targets to internal colleagues, but digital platforms have broadened this scope to a wider social sphere. We show the psychological consequences of this expansion, which invites future studies to explore how other digital behaviors influence organizational outcomes. Finally, we provide a perspective on how cultural nuances frame these perceptions. While our results in the Chinese context are significant, they also highlight the importance of investigating how cultural norms regarding group identity influence the sensitivity to social expectation pressure, inviting future research to conduct comparative studies across different cultural settings (Ma et al., 2025).

### 5.3 Practical implications

The empirical results offer clear guidance for human resource management, particularly within the e commerce sector where digital connectivity is exceptionally high.

Managing the Comparison Environment: Given that upward social comparison mediates over half of the relationship between social pressure and turnover, organizations must focus on managing the internal social climate. HR departments should implement recognition systems that celebrate diverse forms of success rather than just material or performance based benchmarks. By highlighting varied career milestones, companies can reduce the tendency for employees to rely on a single, narrow digital standard of success. This approach aligns with the need to foster a sense of belonging and social recognition within the workplace (Karacay et al., 2022).

Enhancing Career Predictability and Transparency: Our data implies that external pressure is most damaging when internal career paths are unclear. To counter the allure of external peer achievements, firms should provide transparent and predictable career development frameworks. When employees have a clear understanding of their own growth potential and the steps required for promotion, they are less likely to feel that they are falling behind their social peers. This structural clarity serves as a psychological anchor that mitigates the negative effects of social expectation pressure.

Digital Wellness and Career Coaching: Since social media usage duration is linked to these psychological pressures, organizations should include digital wellness in their employee assistance programs. Providing career coaching that helps employees distinguish between curated digital displays and realistic professional growth can empower Generation Z staff to navigate their social networks more rationally. Training programs focused on digital literacy and psychological resilience can help employees manage their comparison tendencies, thereby enhancing retention and long term wellbeing.

Targeted Support for High Pressure Groups: The robustness of our findings suggests that certain employees are more vulnerable to social pressure than others. HR managers can use the insights from our propensity score matching analysis to identify groups that might require additional support. By offering flexible work arrangements or mentorship programs to those in high pressure social environments, organizations can provide a supportive structure that offsets the desire to leave, ultimately improving organizational stability in a competitive market.

### 5.4 Limitations and future research directions

Despite its contributions, this study has several limitations, which also point to promising directions for future research.

First, the study adopted a cross-sectional survey design, which limits the ability to infer causal relationships among variables. Future research could employ longitudinal designs or quasi-experimental methods to more rigorously examine causal mechanisms.

Second, there are limitations regarding sample representativeness. Although the sample included employees from e-commerce firms of different sizes and regions, it was still limited to the e-commerce industry. Therefore, the generalizability of the findings remains to be tested. Future studies could replicate this model in other industries to assess its broader applicability.

Third, the study relied entirely on self-reported data, which may be subject to social desirability bias. Future research could incorporate objective behavioral indicators (e.g., actual turnover records, social media usage logs) and peer or supervisor ratings to enhance measurement validity.

Fourth, the study focused primarily on upward social comparison as the mediating mechanism. However, the psychological processes linking social expectation pressure to turnover intention may be more complex. Future studies could explore other potential mediators (e.g., self-efficacy, career anxiety) and moderators (e.g., social support, organizational culture).

Fifth, although the study used data from a Chinese sample, it did not deeply examine cultural factors. Future research could undertake cross-cultural comparative studies to investigate how cultural contexts, such as individualism versus collectivism moderate the proposed model.

Sixth, the study lacked intervention-based research. While several practical suggestions were proposed, the effectiveness of these strategies remains untested. Future research could design and evaluate targeted interventions aimed at reducing social expectation pressure or reshaping upward comparison tendencies.

## 6 Conclusion

This study explores how social expectation pressure shapes turnover intention among Generation Z employees in the digital age. By applying social comparison theory, we tested a model where upward social comparison serves as the primary mechanism linking social pressure to the intent to leave an organization. Based on a sample of 542 employees, we find that social expectation pressure exerts a direct link to turnover intention. Notably, upward social comparison mediates the majority of this relationship, explaining over half of the total association. These results suggest that Generation Z employees navigate their career paths within a digitally mediated landscape that demands new HR strategies. By identifying these psychological drivers, this study offers a new framework for understanding employee retention and encourages organizations to rethink how they manage the social and psychological wellbeing of their workers.

## Data Availability

The raw data supporting the conclusions of this article will be made available by the authors, without undue reservation.
